# Regenerative medicine: could Parkinson's be the first neurodegenerative disease to be cured?

**DOI:** 10.2144/fsoa-2019-0035

**Published:** 2019-10-10

**Authors:** Mariacruz L Díaz

**Affiliations:** 1 Instituto Nacional de Investigación y Tecnología Agraria y Alimentaria (INIA), Departamento de Reproducción Animal, Madrid 28040, Spain

**Keywords:** clinical trials, DOPAminergic precursors, glycobiology, human embryonic SC, human induced pluripotent SC, human parthenogenetic neural SC, human pluripotent SC, neurodegeneration, Parkinson's, prion-like diseases, regenerative medicine

## Abstract

Parkinson's disease is one of the most insidious neurodegenerative diseases in developed countries. Today, human pluripotent stem cells are produced from embryonic or adult cells, multiplied, differentiated into neural cell lines and ultimately transplanted into disease animal models or patients. Nowadays, DOPAminergic neurons derived from human pluripotent stem cells and human parthenogenetic cells are being clinically tested in China and Australia, respectively. More importantly, good manufacturing practices have been developed and the neurons obtained have been successfully tested in nonhuman primates by teams in Europe, USA and Japan. However, there is a need for translational clinical studies with small molecules tested *in vitro*, as well as testing of the the efficacy of additional therapies.

## Lay abstract

The high prevalence of sporadic cases of Parkinson's disease is a serious health problem in developed societies. A safe regenerative therapy is urgently needed, and is among the most promising treatments. In several years, we can expect to see results from clinical studies using stem cells from across the globe. It is probable that the definitive cure will require more than one therapeutic approach, as discussed in this review.

## Graphical abstract

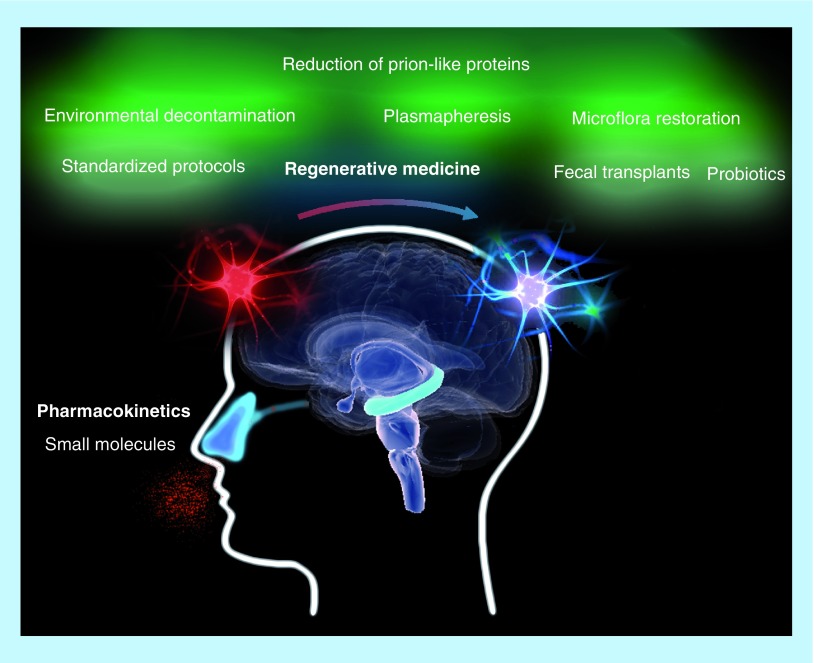

The success of regenerative medicine depends on the restoration of organic homeostasis. Removing prion-like proteins from the body is especially important in sporadic cases of Parkinson's disease, which accounts for 90% of cases. Small molecules identified as enhancers of neural stem cell regeneration should be tested. Reduced doses could potentially be achieved, and unwanted side effects avoided, by a nasal application.

The increasing prevalence of neurodegenerative diseases in developed countries is a fact recognized by the scientific community. Parkinson's disease (PD) is one of the most insidious, and deserves our attention and efforts. It is probable that more than one therapeutic approach will be needed before we unravel the primary/ultimate cause. One of the most promising treatments is based on regenerative therapy. In the 90s, the transplantation of fetal cells in patients with PD aroused great expectation, but drawbacks such as the difficulty in obtaining fetal cells, disparity of results and graft-induced dyskinesias slowed down its use. Later, Yamanaka's attempt to produce an unlimited source of stem cells was a success [1]. He applied a breakthrough procedure by introducing four transcriptional factors (Oct3/4, Sox2, Klf4 and c-Myc) with retroviruses in human fibroblasts. The fibroblasts were transformed into cells with similar potential to embryonic stem cells (ESCs); these were called induced pluripotent stem cells (iPSCs). The iPSCs are capable of self-renewal and differentiation into all three germ layers. Since that publication, there has been an avalanche of work in which iPSCs are induced, multiplied, differentiated into different types of nervous cells and implanted both in patients and in murine disease models. Nowadays, hPSCs can be produced from embryonic or adult cells. Significant advances have been made solving some important drawbacks of Yamanaka's strategy; for instance, derived neurons reach functional maturation preventing formation of tumors; the production efficiency has been increased by millions of cells; and transcriptional factors have been reduced, to be substituted by effective small molecules (SMs). However, to date, it is with human embryonic and fetal cells that best clinical outcomes have been achieved, but their use has also been restricted because the extraction process is really complex and it entails important ethical objections. Here a ground-breaking approach must be mentioned that has been developed with human parthenogenetic cells. Cells are derived from human nonfertilized oocytes whose second meiosis is halted resulting in diploid cells [2]. These diploid cells are derived into parthenogenetic neural stem cells (pNSCs) with only maternal chromosomic material. Currently, DOPAminergic neurons derived from parthenogenetic pluripotent stem cells (pPSCs) are being tested in clinical trials in Australia [3]. It is too early to know what the results will be; although it could open new research avenues, we still have to learn and see the long-term parthenogenetic cell evolution [4].

For centuries, it was believed that there were no neural progenitor cells (NPCs) in the human CNS. Today, it is clear that in the subventricular zone of the lateral ventricle and the subgranular zone of the hippocampus, there are NPCs that could regenerate the nervous cells and be manipulated ‘*in vivo’* with regenerative purposes [5]. Moreover, it has recently been proven in mice that ependymal cells of the fourth ventricle also have neural stem activity; this supports the idea that the entire lining of the CNS probably has dormant NPCs [6]. Regardless, in addition to fetal and embryonic cells, NPCs are the most studied and promising cells up to date. Theoretically, NPCs have almost unlimited multiplication capacity and are multipotent; that is, they are able to differentiate into several types of nerve cells.

Although this overview will focus on the status of regenerative medicine regarding its application in PD, we should be aware that the available information supports the hypothesis that most insidious neurodegenerative diseases (Alzheimer, Parkinson, Amyotrophic Lateral Sclerosis [ALS], etc) share a common pathogenesis. In most of them, at least one protein with abnormal structure is implicated, (for instance β-amyloid in Alzheimer, α-synuclein in Parkinson and superoxide dismutase in ALS) and cell-to-cell transmission follows the paradigm accepted for prion diseases [7]. That is why they are so-called ‘neurodegenerative prion-like diseases’. In some instances, more than one protein is implicated (for instance α-synuclein and tau or β-amyloid and tau). It should be noted that the spectrum is growing, including diseases such as depression. Focusing on synucleinopathies, Prusiner, in 2015, proved that multiple system atrophy (an α-synucleinopathy frequently misdiagnosed as PD) is the second prion disease in humans [8]. Moreover, the sporadic cases account for 90% of all neurodegenerative clinical cases, emphasizing how important it is to consider additional therapeutic approaches besides the regeneration of the damaged tissues.

## Multiplication & differentiation of NSCs

Compared with other tissues, the nervous system has very limited capacity of regeneration ‘*in vivo’*. Therefore, many studies have been devoted to pursuing the production of NSCs ‘*in vitro’* with regenerative purposes. Due to the extensive scientific literature, we will focus only on relevant studies performed with human cells. Adult cell-derived hiPSCs have been utilized in the majority of research studies, in which the cells later on differentiate into NSCs and finally into mature neurons. The more frequently used cells have been the fibroblast [9], bone marrow mesenchymal stem cells (BM-MCs) [10,11] and umbilical cord cells [9,12]. Nevertheless, other adult cells have also been differentiated such as pericytes of the cerebral cortex, as assayed by Karow *et al.* in Germany [13], ecto-mesenchymal stem cells from olfactory mucosa in France by Nivet *et al.* [14], stem cells from carotid body in Spain by Platero-Luengo *et al.* [15], human urine epithelial cells in China by Cheng *et al.* [16], stem cells from dental pulp by Zhang *et al.* [17] and so on.

Since Yamanaka's procedure publication [1], many advances have been made in order to avoid tumor formation and to obtain mature neural cells. In a study published in 2015 by Bardy *et al.* [18], the authors developed a new culture medium superior to existing media traditionally used for growing and maturing nervous cell lineages. Fully functional glial and neuronal cells were derived from human fibroblasts directly into induced neural cells (iNCs) by using two transcriptional factors, Ascl1 (Mash1) and Ngn2 (Nurr1). The same year, Lee *et al.* were able to obtain directly iNSCs from human blood cells by using a single transcriptional factor (Oct4) and chemical inhibitors. The iNSCs were postderived into five types of NCs: GABAergic, DOPAminergic, astrocytes, oligodendrocyte and, for the first time, nociceptive sensory neurons [12].

Notwithstanding, the crucial step to avoid the unwanted use of retroviruses was taken earlier in 2014 by Cheng *et al.* They derived human epithelial cells extracted from urine into NSCs by using a cocktail of three chemicals (Valproic acid VA, Repsox and CHIR 99021) [16]. From the functional standpoint, these SMs are inhibitors; VA inhibits deacetylation of histones, Repsox inhibits glycogen synthase kinase 3β (GSK3β) and CHIR 99021 inhibits EGF. These cells were called chemically induced neural progenitor cells. They obtained cells with an elevated plasticity and a great potential for neuronal conversion by using these chemicals together with hypoxic conditions (mimicking the physiological conditions of oxygen partial pressure of 5% O_2_ in the CNS). It deserves to be mentioned that this procedure could be applied to other cells of an epithelial nature such as uterine menstrual cells, which have already been transformed into iPSCs using Yamanaka's procedure by Ding *et al.* [19]. In August 2015, the same scientific group obtained the direct conversion of human fibroblasts into mature neuronal cells with four additional SMs: Forskolin, SP600125, GO6983 and Y27632. The majority of these human chemically induced neuron cells (hciNCs) had a glutamatergic nature [20]. In October, they went another important step forward in mice; the direct conversion of astrocytes into four types of neurons: dopaminergic, GABAergic, glutamatergic and motor neurons [21]. It is important to emphasize that astrocytes are very interesting neural cells in terms of regeneration because they actively multiply after brain injury and because they are considered precursor cells of neurons in mammals.

It is clear that the type of starting cells, either fetal or adult, determines the final derived cell type. Whether we use transcriptional factors or chemical inhibitors to dedifferentiate adult cells, the epigenetic memory is specific and determinant of the final result. Regarding this issue, we must mention a suggested multicellular eraser of epigenetic marks, an inhibitor of the BET superfamily proteins (I-BET). The I-BET151 competes with BRD4 (bromodomain-containing protein 4) inhibiting its binding to the super-enhancers of core genes. The results indicate that the addition of I-BET151 in the chemical cocktail resulted in a dramatic increase of functional neurons directly from mice fibroblasts [22]. Therefore, proper chemical SMs acting first as erasers of the epigenetic marks and later as inhibitors of growth factors (EGF, TGF and BMP) or enzymes (protein kinase, GSK3β) could be used locally in such a way that theoretically the regeneration of the required cell could be achieved.

In terms of efficient production, the number of neurons obtained by Haus *et al.* in a developed xeno-free culture system was remarkable. They started with hESCs and isolated CD133^+^/CD34- cells with magnetic sorting. In only 10 days, 300 million hNSCs could be produced and in transplanted mice no teratoma were developed [23].

## Production, transplantation & characterization of precursor DOPAminergic neurons

Parkinson's was first described in 1817 by James Parkinson, and to the pathognomonic location of the Lewy bodies in the DOPAminergic neurons of hippocampus, described by FH Lewy in 1910. Initially, the degenerated DOPAminergic neurons are confined into the sustantia nigra and the striatum of the hippocampus, a relatively small area of the brain which could be easily substituted. However, it has been more than 24 years since the first transplants of fetal cells were performed in PD patients. To date, the best clinical results obtained in humans are still those using human fetal cells from the ventral mesencephalon [24,25]. The most important obstacle has been the acquisition of fetal tissues together with a high variability in the recovery rates and the development of dyskinesias in treated patients (Table 1).

**Table 1.** Advantages, disadvantages and results of neural precursors derived from human fetal, embryonic, reprogrammed somatic cells and parthenogenetic cells.

Cell type	Advantages	Disadvantages	Results
hfventral	First clinical studies made	Ethical concerns Scarce source	Excellent long-term results
hESC	Guaranteed cell source H9WA09 GMP in place to produce cells of quality Detailed description of cell markers	Immunosuppression treatment	Functional recovery No tumorigenicity Variability
hiPSC	Absence of ethical inconvenience Matched HLA is possible No immunosuppression treatment Detailed description of cell markers	Time-consuming and high-cost	Functional recovery No tumorigenicity Variability
hpNSC	Less ethical objections Produce neurotrophic agents	Lack of ‘bona fide’ DOPAminergic markers	Unknown long-term evolution
iNC	No pluripotency stage needed	Unknown	Unknown

hpNSC: Human parthenogenetic neural stem cell; hESC: Human embryonic stem cell; iNC: Directly induced neuronal cells from skin cells.

Optional sources have been sought; among the most promising ones are the precursors of DOPAminergic neurons derived from hPSCs, either from reprogrammed adult cells or from ESCs of early blastocysts. Thanks to many excellent studies, several protocols have been published by which precursor DOPAminergic neurons are produced with high efficiency and proper functionality from hESCs [26,27].

Up to now, fetal cells and hESCs have been used under strict management rules in top research centers, and only in those countries with permissive rules. The derived precursor DOPAminergic neurons from hESCs have been the most studied and the best characterized. We have seen that the line designated H9 (Scottish Centre for Regenerative Medicine, UK) or WA09 (WiCell University of Wisconsin Madison, WI, USA) has been deeply studied [26,27]. It has already been approved by the NIH (MD, USA) and it complies with European rules (National Institute for Biological Standards and Control, UK Stem Cell Bank); even a commercial brand offers this H9WA09 line. Theoretically, the unlimited production of precursor DOPAminergic neurons could be possible using these hESCs lines. Therefore, if no more embryos are needed one could wonder what ethical objections there could be in expanding and differentiating the hPSCs into precursor DOPAminergic neurons.

Fortunately for patients with sporadic PD, the first estimations of the number of cells that need to be replaced are very low, approximately ‘hundred thousand’ cells would be sufficient for one patient. Obviously, these numbers are low for ‘*in vitro’* culture systems and few laboratories would be required to cover the needs of PD patients in each country.

Regarding DOPAminergic precursors derived from hiPSCs, the results of preclinical studies made in a monkey model for PD were published in 2017. Takahashi *et al.* [28] reported that the DOPAminergic cells survived for at least 2 years and formed connections with the monkey brain cells. In the treated monkeys, there was a gradual onset of motor functions and no tumor formation was reported. However, the results in terms of survival of DOPAminergic, neurons were highly variable, suggesting that there are still unknown factors in the hiPSC lines that should be characterized before clinical trials can be made.

We are carefully looking at the ongoing clinical trials worldwide (Table 2). In China, they have recruited PD patients for the transplantation of matched human leukocyte antigen (HLA) cells derived from hESCs. In Australia, ISCO started clinical trials with DOPAminergic neurons derived from human parthenogenetic cells. Some drawbacks have been pointed out regarding the use of parthenogenetic cells: these cells express PAX6 factor which have not been identified ‘bona fide’ ventral midbrain progenitors DOPAminergic neurons [27,28]. There are great expectations ahead, but the hurry and commercial interests need to be put in second place when the hopes of millions of patients are at stake. We hope to see PD be the first neurodegenerative disease to be cured thanks to regenerative medicine.

**Table 2.** Ongoing clinical trials worldwide.

Cell type	Clinical trials	Country/Leaders	Consortium/Company/University
hfC hfC	NCT001898390 2010-2021 NCT03128450 2017	UK, Sweden Dr Barker RA, Dr Parmar M China Dr Chun-Feng Liu	TRANSEURO GFORCE Second Affiliated Hospital of Soochow University
hESC	NCT03119636 2017-2020	China Dr Qi Zhou	Chinese Academy of Sciences
hiPSC	R000038278 2018	Japan Dr Jun Takahashi	Center for iPS Cell Research and Application, Kyoto University
UCMSC	NCT03550183 2018	China Dr Shengjun An	Newtherapy BIo-Pharma Technology Co., Ltd Shijiazhuang, Hebei
ASC	NCT02184546 2014-2018	USA	StemGenex, San Diego, CA
BMSC	NCT01446614 2011-	China	Hospital Guanjzhou, Guanjdong
BMSC	NCT03297177 2016-	USA MD Stem Cells	The Healing Institute Margate, FL, USA Euro-Arabian Hospital Dubai, Sharjah, United Arab Emirates
BMSC	NCT02611167 2017-2019	USA Dr Mya Schiess	The University of Texas Health Science Center at Houston Houston, TX
hpNSC	NCT02452723 2016-2020	Australia, Royal Melbourne Hospital, Parkville	International Stem Cell Corporation, Carlsbad, CA

Source NIH https://clinicaltrials.gov/

ASC: Adipose stem cell; BMSC: Bone marrow mesenchymal stem cell; hfC: Human fetal cell; hpNSC: Human parthenogenetic neural stem cell; hESC: Human embryonic stem cell; UCMSC: Umbilical cord mesenchymal stem cell.

## Rejection of transplanted cells

Single cell transcriptome studies have recently revealed that human fetal cells extracted at 16–18 weeks of gestation are not rejected in part due to the absence of the major histocompatibility complex MHC type I. Unfortunately, the results of this study imply that the CNS is not as immunologically inert as we thought, and that ESCs hPSCs could be very distant from adult neurons [29]. The union of the NSCs with the extracellular matrix of the recipient patient, without any doubt, must be important in the rejection or not of the donor NSCs. The extracellular matrix is in contact with the glycocalyx of NSCs and very little is known about how these components are associated in the human brain. Studies concerning glycoconjugates (glycoproteins or glycolipids) of human NPC's glycocalyx are almost absent.

The important role of the chemical structure of carbohydrates in the CNS is illustrated by experiments made in chickens with intracerebral injections of 2-deoxigalactose. The 2-deoxigalactose induces amnesia, which is reverted when coinjected with galactose [30]. As far as we know, only in rodents have the patterns of specific glycans in CNS been studied. All types of neuronal cells in SG and SV zones could be identified with labeled lectins [31]. In fact, also by using lectins, Hamanoue *et al.* isolated NPCs and deciphered the roles of N-acetylglucosamine and its enzymes on NPC functions, such as cellular migration and immunity [32,33]. Taken together, all these facts stress how important the implication of glycoconjugates and their carbohydrates are in the interneuronal connections of the CNS.

Therefore, if we want to advance into successful tissue replacements more glycobiology studies will be needed to support future glyco-engineering approaches [30].

## From bench to bed

There are translational prospects for neurodegenerative diseases, for example, from the studies made in China. The astrocytes localized in the SG and SV zones behave as neurogenic precursor cells in adult mammals [34]. This peculiar characteristic makes them ideal targets for regenerative use *in vitro* and *in vivo*. In order to convert astrocytes into neuronal cells, Cheng *et al.* dissected the essential chemicals used in their initial work from three (valproic acid, Repsox and CHIR 99021) to two SMs (valproic acid and Repsox). Then, they skillfully replaced SMs by drugs already authorized, which should simplify the authorization process for the new pharmacological indications. Valproic acid, an antiepileptic drug (inhibitor of histones deacetylases), and Tranilast, an antiallergic drug (inhibitor of EGF), were used ‘*in vitro’* with successful results in astrocytes of adult mice [21]. The authors suggested the possibility of applying these chemicals directly in patients with PD. From a translational perspective, this could be a ‘from bench to bed’ study. Therefore, we would expect that the local treatments with valproic acid and tranilast could favor the differentiation of human astrocytes into neurons whenever required.

We have to point out that the use of valproic acid in patients with PD could add other advantages apart from its important antidepressant therapeutic effect, that is, its positive effect on the expression of galectin-1 [35]. Galectin-1β (galactose-binding lectin) is known as a neuroprotective agent and promotor of the axonal regeneration [36], emphasizing again the important role that the galactose and galectin-1 could have in the functional connection between neurons and extracellular matrix. On the other hand, strong evidences support that α-synuclein is propagated by exocytosis–endocytosis from cell to cell. Importantly, the receptor implicated in the endocytosis of pathogenic α-synuclein fibrils (N-methyl-D-aspartate receptor NMDA receptor) was competitively inhibited by galectin-1 in hNSCs. In fact, it has been proven that human BM-MCs produce galectin-1 which, in turn, behaves as a competitive protein with α-synuclein in cocultured hNSCs. Moreover, in a parkinsonian mouse model, galectin-1 improved neuron survival and motor function [37]. Studies in Alzheimer's models, both *in vitro* and *in vivo* have also supported the role of the NMDA receptor in the cellular internalization of β-amyloid fibrils. In truth, both increased β-amyloid clearance and survival of hippocampal neurons were seen when BM-MCs were injected in the tail vein of mice [38], probably through the same mechanism described for α-synuclein. Therefore, in α-synucleinopathies as well as in diseases with β-amyloid fibrils formation, pharmacokinetics studies of SMs deserve to be conducted.

Chronic systemic treatments with VPA have reported some worsening of Parkinson's symptoms. These cases are reversible when the treatment ceases. Therefore, to undertake pharmacokinetic studies of local application would allow decreased doses, the overcoming of unwelcome secondary effects and, hopefully, the promotion of NSCs present in the hippocampus. In order to apply the treatments with SMs, one of the best routes could be through the nose. Anatomically, the olfactory bulb is very close to the roof of the nasal cavity and functionally, it is connected with the hippocampus. In a recent study made by Alvarez-Buylla *et al.*, the authors discovered that in the subventricular zone of the lateral ventricle, the NSCs or type B1 cells divided symmetrically giving rise to an asymmetric population of 20% self-renewal NSCs and 80% differentiated neurons [39]. We wonder if we could reverse these percentages in PD patients. We also know, thanks to the work made by Chen *et al.* in 2017, that the specific inhibition of GSK3β favors NSC self-renewal while the inhibition of GSK3α favors neuronal differentiation in mouse ESCs [40]. Therefore, if this holds true in humans, it could be possible to use nose instillations of GSK3β inhibitors or other SMs in order to increase the number of NSCs. To end this exposition on the state of the art of nervous tissue replacement, the good news is that the results of the transplantation of NSCs derived from human fetuses into Parkinson's patients have been more than encouraging. 24 years after implantation of fetal ventral cells, which were injected in the putamen, there was a complete recovery of the innervation with still functional dopaminergic neurons. Motor symptom recovery and withdrawal of the treatment with LDOPA resulted, and following deaths were not owing to the disease [41,42].

## Future perspective

Today, human ESCs are becoming available in many countries around the world [43–45]. Agreements on robust protocols for multiplication and differentiation of ESCs and iPSCs, good practice codes and standards, disclosed specs for the starting somatic cells, and robust cryopreservation procedures of cells [45,46] and so on, should be reached as soon as possible. This will assure a fruitful outcome for the enormous efforts that are being made in this research field around the world. In fact, a few European countries together with USA and Japan have formed a GForce-PD consortium, which has already begun working in this direction and will start very soon with human clinical trials [47].

We must not forget that α-synuclein prions exist in patients with multiple system atrophy, that α-synuclein is present in organic fluids and in other parts of the body besides the brain, and finally, that in some patients other prion-proteins could coexist. Therefore, we should take important sanitary actions because prion proteins do not respond to standard decontamination procedures. Generally, in laboratories and hospitals around the world, standard conditions used for sterilization in an autoclave are 120°C for 20 min; these are not enough to decontaminate prions. Therefore, we strongly recommend strict protocols for decontamination of prion-like protein, especially in the use of surgical material which should be new for each patient in clinical trials.

Some other clinical therapies have been suggested such as the reduction of the levels of pathological proteins with plasmapheresis [48] or the restoration of intestinal leaking with fecal transplants [49,50]. Although ideally these treatments should be done before regenerative therapy, unfortunately little or nothing is known about their efficacy and safety. If we do not take into consideration all these precautions, transplanted cells could be negatively affected by the presence of prion-like proteins in the recipient patients, endangering their recovery or masking good results.

There is a fine compromise between ‘rejection’ and ‘teratoma formation’; the less differentiated a cell is, more probability exists that a teratoma will develop yet there is a lower probability of rejection. Regarding ‘teratoma formation’ many technical advances have been made since Yamanaka's first studies. Almost all scientists already claim that their cells have not developed tumors in long-term trials. To circumvent the ‘rejection’ either an immunosuppressive treatment should be applied or compatible grafts should be used. Almost all clinical studies have included an immunosuppressive treatment, except in the trial made with autologous iPSC from which we still do not know the results.

Unfortunately, there is still a high variability in the recovery rate in the studies made with DOPAminergic precursors derived from ESC and hiPSC (Table 1). Nowadays, autologous hiPSCs derived from patients with genetic Parkinson's cannot be used. Hopefully, in the near future, different types of stem cells will be produced and used in agreement with the pathogenesis of PD.

Certainly, there is still a way to walk before nervous tissue replacement therapies are fully implemented in daily clinical practice. Meanwhile, ‘*in vitro*’ studies should focus on the key elements for human embryonic and fetal cell transplants to be successful. In the case of PD, the joint efforts of many researchers from around the world are bringing us closer.

**Executive summary**

- Different strategies for obtaining neural stem cells are being tested worldwide.
- It is probable that a cure will need more than one therapeutic approach.
- Pharmacokinetic studies should be performed with small molecules.
- Additional therapies (for example fecal transplant, microflora reconstitution) could help to restore endogenous physiological functions.
- Protocols of prion-like proteins decontamination should be thoroughly applied.
